# Spectral energy balancing system with massive MIMO based hybrid beam forming for wireless 6G communication using dual deep learning model

**DOI:** 10.1016/j.heliyon.2024.e26085

**Published:** 2024-02-14

**Authors:** Ramesh Sundar, Mohammad Amir, Ranjith Subramanian, D. Prabakar, Jayant Giri, G. Balachandran, Furkan Ahmad

**Affiliations:** a Department of Networking and Communications, School of Computing, Faculty of Engineering and Technology, SRM Institute of Science and Technology Kattankulathur, Chennai, India; b Department of Electrical Engineering, Indian Institute of Technology (IIT), Delhi, India; c Department of Electronic and Communication Engineering Jeppiaar Engineering College, Chennai, India; d Department of Data Science and Business Systems, SRM Institute of Science and Technology, Kattankulathur, Chennai, India; e Department of Mechanical Engineering, Yeshwantrao Chavan College of Engineering, Nagpur, Maharashtra, India; f Division of Sustainable Development, College of Science and Engineering, Hamad Bin Khalifa University, Qatar Foundation, Doha, Qatar

**Keywords:** MIMO, Dual deep network, DDN, Beamforming, Hybrid network architecture, Energy balancing, mmWave, Analog precoder, Digital precoder, 6G communication

## Abstract

This work aims to provide an effective hybrid beam forming method with Dual-Deep-Network to overcome overhead for mm-wave massive MIMO systems. In this paper, a Dual-Deep-Network technique is described for the extraction of statistical structures from a hybrid beam forming model based on mmWave logics, as well as training logic for the network map functions. The proposed approach of DDN is trained with proper data sequences used for communication and the training phase is conducted with the norms of numerous channel variants. With the nature of diverse channel states, a Dual-Deep-Network is required to manipulate the level of presence and abilities even after training as well. The performance level improvements are practically summarized in both the transmission and reception entities with the help of the proposed hybrid network architecture and the associated Dual Deep Network algorithm. Specifically, the BER versus SNR and spectral efficiency versus SNR are evaluated as well as the resulting accuracy levels are cross validated with numerous classical communication techniques. This paper shows the processing difficulties of the proposed approach and typically cross-validates with other beam forming logics. The computational cost and performance estimations are improved, and the metrics are clearly visualized on this paper based on improved beamforming procedures as well as the proposed approach of DDN based Multi-Resolution Code Book performance metrics are estimated clearly with proper mathematical model investigations. With 7Kbits/s/Hz and 1e-1, respectively, the key metrics of spectral efficiency and BER are enhanced.

## 1 Introduction

Revolution on wireless communication system have traditionally undergone for every decade. We are now looking for 6G that is lying in the analysis level, through system agreement headed for viable roughly 2020 among extensive implementation in 2030. The current marketplace requires 6G and it should sustain elevated system ability as compared with present 5G methods [1]. To achieve high system capacity, we must focus on key factors of Dense Networks (DN), Higher Bandwidth slots and high Spectral efficiency [2]. One of the most common challenges that come to mind is the problem of propagation losses, which are higher at very high frequencies. Millimeter-Wave Massive-MIMO provides a clever approach to combine all these key factors and increase the high data rate network speed [3].

Now-a-days, each and every network organization requires to improve the throughput level for providing drastic communication to clients as well as handle the related wireless network traffic scenarios. The enhanced MIMO strategies attain 10-fold enhancements on throughput level via effective improvements in spectral energy balancing with respect to bit/s/Hz/cell during the appliance of similar network density and bandwidth ratios of the base stations as in the current network scenario. The astonishing and amazing benefits are attained through furnishing the base stations by groups of several-antennas to facilitate spatial level multiplexing of variant user-mortals and beam forming more effectively [4]. These techniques or technologies can be implemented in different antenna formats, and we would explore two of the antenna arrangements using the premise after the Massive-MIMO expertise as well as the associated execution connected intends strategy. The design and the development of wireless systems is difficult and most importantly the Radio Frequency (RF) antenna system design is most challenging.

Conversely, the plan of proposed beam forming template be inhibited through costly millimeter Wave radiofrequency (RF) sequences. Conventional completely digitalized beam former desires toward a radiofrequency sequence for each antenna aspect and therefore enforce fanatical energy utilization as well as implementation expense. A new technique is required to resolve such mentioned issues, so that Hybrid Beam forming (HBF) Logic is introduced in this paper [5], in which it accumulates the huge information sequences with proper beam forming accuracy by means of Millimeter-Massive-MIMO in association with combined Pre -coder logic [6]. Some numerical algorithms are implemented in last years to obtain feasible hybrid pre-coding such as manifold optimization (MO) [7],

Karush-Kuhn-Tucker (KKT) [8] and orthogonal matching pursuit (OMP) [5] based algorithms. To accomplish optimal performance of HBF and reduction of time consumption Deep Learning based algorithms were proposed [9,10]. For hybrid pre-coding matrices designing, we need a precise assessment of Millimeter Wave-channel and an exigent task due to the highest quantities of antenna ratios at both the transceiver ends. This leads to elevated training model transparency as well as constraints on the RF chains [11,12].

The hybrid beam forming structure used a switched beam forming strategy [13], where the beam space is denoted by a codebook of numerous code words, because Millimeter-Wave channel is scarce in the slant field. Searching through their individual codebooks yields the leading transmit/receive beams. There are two types of codebook design, which are referred to as low resolution (coarse sub-codebook) and high decision factors (excellent sub-codebook), respectively. Both types wrap the intended slant and are described by large numbers of well-beams [14]. Here we are considering both types of codebooks which increases the adaptability of the system design. In Ref. [15] a novel beam forming logic is proposed with adaptive multi-dimension codebook concept for mm Wave schemes, in which the code words produced the beams with respect to the spatial beam width area. In Ref. [16] literature, a novel Discrete-Fourier-Transform with phase shifting principles are introduced with respect to codebook formulations to improve the beam forming ratio by reducing the variations in beam width and the associated distance elevation ranges. These procedures are not producing constant probabilistic results, based on the number of clients the training process scaling ratio differs [31,32].

The multi resolution codebook-based hybrid beam forming using the Dual Deep Network (DDN) method is designed to solve these mentioned limitations. In which DDN Multi resolution code book-based beam forming is espoused for the perception of Millimeter-Wave Massive-MIMO scheme instead of conventional methods for eliminating the energy wastages and enhance spectral competence, Less Overhead hybrid beam forming is proposed. In particular, a multi-resolution code book based on DDN, consisting of the training phase and the beam forming phase. Using the algorithms of Enhanced Orthogonal Modulation (EOM) Pre-coding and Gram-Schmidt Procedure (GSP), we are designing the analog and digital pre-coding matrix in the training phase [31,32]. This method integrates novel codebook logic with the hybrid pre-coding dual state pre-coding methodology. We train those values in the Dual Deep Learning Network, which has the CNN-Hybrid architecture, after the MR codebook has been predicted. The design of a multi-level beam sequence that correctly allots a codebook with a fixed signal power to the training phase is taken into consideration in this study. A subset of the available code books is chosen for an increased mean data rate within a constrained time frame thanks to a number of multi-resolution code books [17,18]. To further reduce the overhead of training phase we proposed the DDN for code book design. Dual Deep Network (DDN) is the deep learning model, in which we are implementing the DNN architecture as hybrid structure. The two architectures of DNN are created for the configurations of MR code book. By using these two architectures in hybrid way, we can get a fastest and effective beam forming with less overhead of training phase.

The remainder sections are arranged in the following structure: Section-II describes the mm Wave massive MIMO scheme with channel modelling. The further section, Section-III initiates the methodology of the proposed algorithm DDN and detailed description of each algorithm for EOM based Hybrid Beam forming and multi resolution codebook based DDN integration is discussed. In Section IV, we present experimental results of the system, followed by a discussion on performance comparison with existing schemes. At last, Section-V terminates this paper with proper conclusion.

## 2 Methodology

### 2.1 System architecture of Mmwave massive MIMO

In this paper, a new Millimeter Wave Massive MIMO scheme is introduced with a Base station includes a Uniform-Linear-Array (ULA) based transmitter antennas (N_t_) as well as the Client receiving antennas (Nr). In this approach, a Base station forwards the unconventional data packet streams (Ns) to the receiver end with the consideration of all such communication-links have no relevant metadata [19]. In addition processes are considered that the Base station as well as the client contains N^t^_RF_ and N^T^_RF_ radio-frequency chains with respect to the following constraints such as Ns ≤ N^t^_RF_ ≤ Nt and Nt ≤ N^T^_RF_ ≤ Nr. Apart from this, a popular Saleh Valenzuela (SV) channel metric is considered over this approach and the associated channel metric H is specified in the form of Eq. (1).

(1) $$H=\sqrt{\frac{N_{t}N_{r}}{N_{p}}}\left(\alpha_{0}a_{t}\left(\theta_{0}^{t}\right)a_{r}\left(\theta_{0}^{r}\right)+\sum_{p=1}^{P}\alpha_{p}a_{t}\left(\theta_{p}^{t}\right)a_{r}\left(\theta_{p}^{r}\right)\right)$$

$N_{p}$ shows the amount of NLoS. In addition, the guiding-vectorsS_t_ and S_r_ are characterized as the exhibit reactions at the Base station as well as the client accordingly. Besides $\theta_{p}^{t}$ and $\theta_{p}^{r}$ address the point of departure at the basestation in association with the point of arrival at the client, individually. To portray a Uniform-Linear-Array, $s_{t}\left(\theta_{p}^{t}\right)$ ϵCN_t_ ×1 and $s_{r}\left(\theta_{p}^{r}\right)$ ϵ CN_r_ ×1 can be expressed as Eqs. (2), (3).

(2) $$s_{t}\left(\theta_{p}^{t}\right)=\frac{1}{\sqrt{N_{t}}}{\left[1,e^{-j2\pi \frac{d}{\lambda }\sin\;\theta_{p}^{t}},\ldots \ldots \ldots ,e^{-j2\pi \frac{d}{\lambda }\left(N_{t}-1\right)\sin\;\theta_{p}^{t}}\right]}^{T}$$

(3) $$s_{r}\left(\theta_{p}^{r}\right)=\frac{1}{\sqrt{N_{r}}}{\left[1,e^{-j2\pi \frac{d}{\lambda }\sin\;\theta_{p}^{r}},\ldots \ldots \ldots ,e^{-j2\pi \frac{d}{\lambda }\left(N_{r}-1\right)\sin\;\theta_{p}^{r}}\right]}^{T}$$

The term ‘d’ is assumed at this point that the antenna space specification is characterized by 'λ' during the frequency of a carrier recurrence in which the term H has low positions. Since the restricted dissipating features in the Millimeter-Wave specific huge MIMO channel metric, demonstrating that close ideal throughput is accomplished by utilizing restricted measures of radio-frequency chains.

At that point, high-dimensional simple pre-coder P_A_ is assumed with lowest RF chain using EOM architecture as well as a squat aspect digital pre-coder P_D_ using Gram-Schmidt Procedure (GSP), then a hybrid decoder be indicated like P←P_A_P_D_. Hence, the specific signal for transmission T is given as Eq. (4).

(4) $$T=P_{x}=P_{A}P_{D}x$$

where x indicates the base signal amid standardized supremacy E[x] = I*N_s_, and the tr{${P_{A}}^{H}$} ≤ N_s_ is assumed to assure the constriction of transmission energy level. Consequently, the accumulated signal distances and associated levels are described as Eq. (5).

(5) $$y=C^{H}H_{x}+C^{H}n=\left(C_{D}^{H}C_{A}^{H}\right)HP_{A}P_{D}x+C_{D}^{H}C_{A}^{H}\;n$$

where 'n' is a hybrid combiner, in which C_D_ and C_A_ are delineated as a digital-combiner and analog-combiner, is an Additive White Gaussian Noises (AWGN) as well as ${C_{H}=C}_{D}^{H}C_{A}^{H}$. Reminder that the analog precoder/combiner is forevermountedthrough analog-phase shifting law as well as whole P_A_ and C_A_ constituents should comply as Eq. (6).

(6) $$\left|{\left\{P_{A}\right\}}_{i,j}\right|=\frac{1}{\sqrt{N_{t}}},\left|{\left\{C_{A}\right\}}_{i,j}\right|=\frac{1}{\sqrt{N_{r}}}$$

With the MIMO massive mmWave system, the full use of the mmWave channel sparsity can significantly improve the performance of hybrid precoding.

### 2.2 Proposed DDN based multi resolution codebook for hybrid beamforming

The formation characteristics of such Millimeter-Wave systems are not exploited by conventional methods; historically, during expense of degrading hybrid pre-coding of schemes, low-complexity systems are carried out.

Therefore, these problems are not fundamentally solved by previous works and new strategies require moving urgently to increase the hybrid-precoding effectiveness of the massive Millimeter-Wave MIMO. The objective of this research is to provide an efficient precoding technique for mmWave massive MIMO with deep learning. An extremely extraordinary procedure to manage volatile information and solving complex non-linear issues have recently been the.

An emerging approach is called deep learning. Therefore, this work examines a framework that incorporates deep learning in mmWave MIMO systems in hybrid precoding. Analog precoding was adopted on the basis of Enhanced Orthogonal Modulation (EOM) [20]. For the mmWave MIMO systems, hybrid energy-efficient precoding is proposed where EOM analog precoding is adopted to reduce energy consumption and to increase energy efficiency in place of phase-shifters. In particular, the EOM, made up of delay lines and switches, allows simultaneous monitoring of phases and amplitudes, thereby imposing additional restrictions on analog precoding [31].

To demonstrate how a hybrid precoder can map the nonlinear operation, a new DDN-based system is designed. Next, a new training policy has been created to improve DDN effectiveness. The computational complexity model for the suggested methodology is shown together with that for other common precoding techniques. Digital analysis is used to examine the output of the proposed DDN-based mmWave massive MIMO system. In particular, the Bit Error Rate is assessed using different study rates and batch sizes, and the training dataset's performance is contrasted with that of many widely used techniques [[21], [22], [23], [24]]. In addition, 4500 simulation iterations were used to train the network. We displayed findings for the hybrid precoding scheme based on DDN's SNR in terms of spectrum efficiency. The figure displays the whole block diagram.

A EOM Analog Precoder Algorithm

In mmWave MIMO systems, the number of RF chains must always be substantially less than the number of antennas in order to save costs, power consumption, and complexity. Sadly, a decline in RF chains can unavoidably lead to capacity loss. The next issue is how many RF chains are required to perform at least as well as the ideal fully digital precoder. To achieve the maximum spectrum efficiency possible with full digital precoding, the proposed EOM precoding only requires the same number of RF chains as data streams., i.e., $N_{RF}\geq N_{s}$. First, the following assumptions are employed:

The columns of are orthogonal to each other, i.e. Eq. (7),

(7) $$G_{BB}G_{BB}^{H}=\gamma^{2}I_{N_{RF}}$$

Where γ is a constant. Then, the optimization problem can be reformulated as Eq. (8).

$$\log_{2}\left(\left|I_{N_{r}}+\frac{\rho \gamma^{2}}{N_{s}\sigma^{2}}\;HG_{RF}G_{RF}^{H}H^{H}\right|\right)$$

(8) $$s.t.\;G_{RF}\in F$$

The respective function in can be further expressed as Eq. (9).

$$\log_{2}\left(\left|I_{N_{r}}+\frac{\rho \gamma^{2}}{N_{s}\sigma^{2}}\;HG_{RF}G_{RF}^{H}H^{H}\right|\right)$$

(9) $$=\log_{2}\left|Z_{k}\right|+\log_{2}\left|1+\frac{\rho \gamma^{2}}{N_{s}\sigma^{2}}G_{RF}^{\left(k\right)H}W_{k}G_{RF}^{\left(k\right)}\right|$$

Where $C_{k}$ is a semi-definite matrix defined by Eq. (10), (11), (12).

(10) $$Z_{k}=I_{N_{RF}-1}+\frac{\rho \gamma^{2}}{N_{s}\sigma^{2}}{\left({\underline{G}}_{RF}^{\left(k\right)}\right)}^{H}H^{H}H{\underline{\;G}}_{RF}^{\left(k\right)}$$

(11) $$W_{k}=H^{H}\;H-\frac{\rho \gamma^{2}}{N_{s}\sigma^{2}}H^{H}H\;{\underline{G}}_{RF}^{\left(k\right)}Z_{k}^{-1}{\left(G_{RF}^{\left(k\right)}\right)}^{H}H^{H}\;H$$

(12) $$O_{\max}=\{\sqrt{\frac{1+{\left(\tan\;\;\tan\;\omega \;\right)}^{2}}{{\left(1+\tan\;\;\tan\;\omega \;\right)}^{2}}},0<\varphi_{1}\leq \frac{\pi }{2},\pi <\varphi_{1}\leq \frac{3\pi }{2}\;\sqrt{\frac{1+{\left(\tan\;\;\tan\;\omega \;\right)}^{2}}{{\left(1-\tan\;\;\tan\;\omega \;\right)}^{2}}},\frac{\pi }{2}<\varphi_{1}\leq \pi ,\frac{3\pi }{2}<\varphi_{1}\leq 2\pi$$

For convenience, how to obtain the analog precoder is provided in Algorithm 1 below.

● Algorithm 1: Enhanced Orthogonal Modulation Precoding

Inputs: H, $N_{RF},N_{s},N_{t},I_{\max}$ and Output: ${\;G}_{RF}$.

1. Initialize $G_{RF}$.
2. For i = 1: $I_{\max}$.
3. For j = 1: $N_{RF}$.
4. Calculate $Z_{j}$ and $W_{j}$ using Eq. (10), (11).
5. For x = 1: $N_{t}$.
6. Evaluate ${be}^{j\omega }$ for the calculated values of $W_{j}$ and $G_{RF}$.
7. Evaluate $O_{\max}$ using Eq. (12) and ${\;F}_{RF}\left(x,j\right)=O_{\max}$ using Eq. (13)
8. .

(13) $${\;F}_{RF}\left(x,j\right)=O_{\max}e^{jangle\left({be}^{j\omega }\right)}$$

9. end
10. end
11. end
B Multi Resolution Codebook Design

The spatial correlation and angular sparsity, two key mmWave channel features, are intended to be reflected in the codebook's architecture. The dominating eigenvectors of the spatially correlated channel point in what are considered to be ideal directions, in contrast to the general channel, whose eigenvectors are tropically distributed. Additionally, the channel has a tendency to have a small number of dominant eigenvectors due to the angular sparsity [25].

The multi-resolution codebook is designed with N levels and each code word is defined in accordance with the required beam range. One definition of the nth level codebook is Eq. (14).

(14) $$B_{n}\equiv \left\{B_{1}^{\left(n\right)},\ldots \ldots ,B_{\left|w_{n}\right|}^{\left(n\right)}\right\}$$

This codebook will cover the cell homogeneously, like the spatial range of o = (−1, 1).

The codebook with multiple resolutions is built in such a way that $\left|B_{1}\right|<\ldots <\left|B_{N}\right|$, implying narrowerbeams at higher levelsof the codebook with spatial beamwidth, shown in Eq. (15).

(15) $$w_{n}=\frac{2}{\left|B_{n}\right|}$$

Let $o_{n}^{k}$ indicate the spatial period enclosed by ${\;B}_{n}^{k}$. Then we assume, without loss of generality, expressed in Eq. (16).

(16) $$o_{k}^{\left(n\right)}=\left(-1+\frac{2\left(k-1\right)}{\left|B_{n}\right|},-1+\frac{2k}{\left|B_{n}\right|}\right),k=1,\ldots \ldots .,\left|c_{n}\right|$$

In a perfect world, the code word $B_{n}^{k}$ would offer an essentially constant beamforming advantage over $o_{n}^{k}$. The ideal beamforming gain over o nk should be sent by the codeword B n^k. By using the aforementioned multi-level codebook to divide and conquer the N levels of the codebook, training can be accomplished [26]. The receiver typically chooses and feeds back the index among the code words chosen in the (n-1)^th^ training phase that has the highest received signal power in the nth training phase. The aforementioned training steps are repeated until the signal power cannot be increased any further; in all, most training symbols are needed. The strongest beam-to-leakage ratio (BLR), which is the ratio of the expected average power inside the covered beam range to that outside the beam range, is what we describe as a codebook in this section. We also suggest a method for creating a codebook that accounts for the potential interference generated by the beam.

Consider a $N_{t}$ transmit antennas placed in the transmitter, the vector direction to the spatial frequency θ=sinsin(φ)∈(−1,1) with φ∈(−π/2,π/2) indicating the AOD as Eq. (17).

(17) $$t\left(\theta \right)={\left[1\;t_{2}\left(\theta \right)\ldots \ldots .\;t_{N_{t}}\left(\theta \right)\right]}^{T}$$

Where $t_{N_{t}}\left(\theta \right)=e^{j2\pi \frac{N_{t}-1\;\Delta }{\lambda }\theta }$ is the signal phase shift at the N_t_^th^ antenna, Δ is the spacing of the antenna, λ is the carrier signal wavelength.

(18) $$V={\left[v_{0},\ldots ..,v_{M-1}\right]}^{T}$$

Here, In Eq. (18), V is the beam forming vectors of M number of transmit antennas. For the users lying in the spatial interval, the average radiated power as Eq. (19), where $w_{s}$ denotes the beamwidth of $o_{s}$.

(19) $$R=\frac{1}{w_{s}}\int_{\theta \epsilon o_{s}}V^{H}t\left(\theta \right)t{\left(\theta \right)}^{H}Vd\theta$$

The average power leaked by V covering $o_{s}$ can be expressed at the same time as Eq. (20) while BLR is formulated as below in Eq. (21).

(20) $$L=\frac{1}{2-w_{s}}\int_{\theta \epsilon o\backslash o_{s}}V^{H}t\left(\theta \right)t{\left(\theta \right)}^{H}Vd\theta$$

(21) $$BLR=\frac{R}{L}$$

C Dual Deep Learning Network based hybrid Beamforming

Convolution Neural Networks (CNNs), the most common structure of deep learning frameworks, are comparable to multi-layer perceptron's (MLPs). In order to enhance its learning and mapping capabilities (ANN), CNNs have several hidden layers in contrast to traditional artificial neural networks. With the help of activation functions based on the output of these units, the output of each hidden layer of a CNN can be formed. For nonlinear operation, the Rectified Linear Unit (ReLU) function and the Sigmoid function are typically utilized. They are defined respectively as ReLU(x) = max(0, x) and $sigmoid\left(x\right)=\frac{1}{1+e^{-x}}$. Assuming one as the argument and the network output is denoted as *o* and the mapping operation can be presented as *i*, in which it denotes the input data.

(22) $$z=f\left(i,w\right)=f^{\left(n-1\right)}\left(f^{\left(n-2\right)}\left(\ldots .f^{1}\left(v\right)\right)\right)$$

**Fig. 1:**
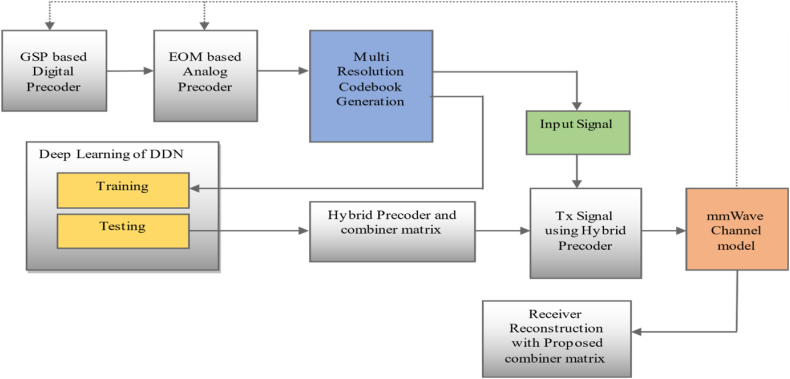
Block diagram of proposed system.

**Fig. 2:**
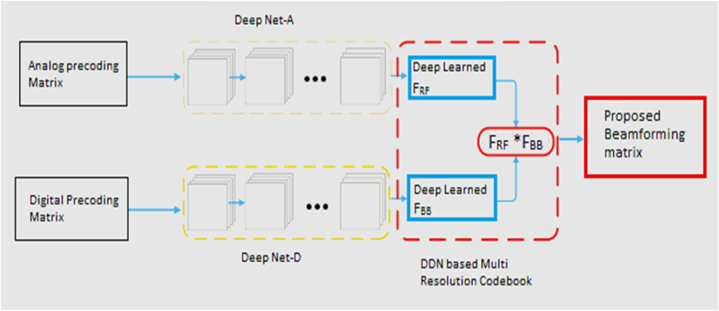
Dual Deep Network based Hybrid beam forming.

**Fig. 3:**
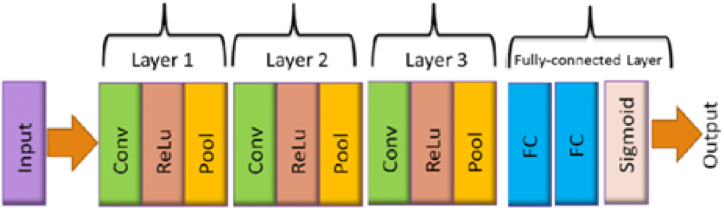
Architecture of deep net.

In Eq. (22), n and w are the number of neural network layers and the weights of the neural network (see Fig. 1). As shown in Fig., we are building a DDN frame for hybrid precoding, as the figures show, Fig. 2 and and Fig. 3. Here, in the input layer, its dimension, a fully connected (FC) layer of 128 units, determines the length of each training sequence in order to capture the features of input data. The next two hidden layers for processing are also FC layers with 400 units and 256 units. In order to disturb the signals with AWGN or other distortion, a layer of noise consisting of 200 units is added to the signal for mixing distortion. We will then design the remaining two hidden layers of 128 units and 64 units in order to achieve decoding. In addition, the output layer is used to generate the expected network output signals.

Furthermore, note that as the activation function of the hidden layers and the input layer, the ReLU function is introduced. A special activation, however, is designed to enforce the power restriction in the output layer, as Eq. (23), while the loss function is written to train the hybrid precoder logic to realize the precoding as Eq. (24).

(23) $$f\left(s\right)=\left(\left(s,0\right),N_{s}\right)$$

(24) $${Net}_{loss}={\left\|G_{1}-G_{RF}G_{BB}\right\|}_{F}=\sqrt{tr(\left(G_{1}-G_{RF}G_{BB}\right){\left(G_{1}-G_{RF}G_{BB}\right)}^{H}}=\sqrt{\sum_{t=1}^{\min\left\{N_{t},N_{s}\right\}}\delta_{t}^{2}\left(G_{1}-G_{RF}G_{BB}\right)}$$

where ||. ||F indicates the Frobenius standard, and G_RF_ and G_BB_ represent an analog precoder based upon EOM from algorithm 1 and the digital precoder based on Gram-Schmidt (GSP) proceedings. δ indicates the matrix's singular values. Then we use the DDN framework to build an auto-encoder that is supplied by Eq. (25).

(25) $$G_{1}=f\left(G_{RF}G_{BB};\Omega \right)$$

f(.) refers to the mapping relationship as equ 23, This offers the complete training process that is described below, and iv is the example dataset. In order to extract structural statistics from the mmWave-based model, we create a training mechanism and see the suggested deep learning scheme as a mapping operation. Deep Net-A and Deep Net-D, respectively, produce random data sequences after initializing G_RF_ and G_BB_ as empty matrices. According to various channel conditions, DDN is trained on the input data sequences, and RA and RD can be updated.

In synchronous form, physical AOA $\theta_{p}^{r}$ and AOD $\theta_{p}^{t}$ may be generated randomly and much iteration allows us to obtain a bias of $G_{RF}\;and\;G_{BB}$ input signals based on DDN output. The training data set Ω consists of the structural features of the massive MIMO mm Wave model, the entry data sequences, and the DDN output. This is an uncontrolled learning strategy. The DDN must be tested after thorough training in the next phase. Without iterations, the optimal $G_{RF}$ analog and $G_{BB}$ digital precoders can be obtained based on the given input signal vectors for each channel condition. The Stochastic Gradient Descent (SGD) algorithm with momentum is then used as a basis of the proposed method to process the loss function,

(26) $$\left\{ \begin{array}{c} G_{RF}^{j+1}=G_{RF}^{j}+b\\ G_{BB}^{j+1}=G_{BB}^{j}+b \end{array}\right.$$

In, Eq (26), for the facilitation of the gradient element, b is denoted as the bias. In addition, the iteration number is denoted as j, and the randomly generated initial solution is assumed to be $G_{RF}^{0}$ and $G_{BB}^{0}$. Specifically, the b update process can be provided by Eq. (27).

(27) $$b=\alpha b-\varepsilon g=\alpha b-\varepsilon \frac{1}{N}\nabla_{G_{RF}{,G}_{BB}}\sqrt{\sum_{i=1}^{\min\left\{N_{t},N_{s}\right\}}\delta_{i}^{2}\left(G_{1}-G_{RF}G_{BB}\right)}$$

where α indicates the momentum parameter and ϵ indicates the rate of learning. Synchronously, g and N are respectively represented by the gradient element and the number of samples. Specifically, in Algorithm 2, the learning framework is defined for super hybrid precoding.

Furthermore, in order to analyze its performance, the Mean-Square-Error (MSE) is introduced to examine the precoding performance of the deep learning-based precoding strategy, which can be given as Eq. (28).

(28) $$MSE=E{\left\|G_{1}-G_{RF}G_{BB}\right\|}^{2}$$

The 'precoder' of the DDN architecture is proposed to predict the indices of the RF beamforming/combining vectors from the receive measurement vector y, as in Fig. 3. This network consists of three convolutionary layers and two fully connected layers with one output layer and is fed by the multi-resolution codebook output described in Section III-B. Activation of Relu and max pooling are followed by each convolutionary layer. There are two fully linked layers in the network; one predicts the transmit beamforming vector indices, and the other predicts receive combining vector indices. The dimensions of the two layers are equal to transmit and receive codebook cardinalities, G and D, i.e., the number of beamforming/combining vector candidates.

This DDN architecture provides the main two advantages: i) optimization of channel estimation in unsupervised manner along with energy efficiency focus and ii) it trains the prediction of hybrid beamforming and combining matrix from the multi resolution codebook generated matrix for multiple channel conditions. Thus, it has less overhead for the training of beamforming.

D. Complexity Analysis

One of the key rewards of the proposed DDN hybrid beamforming is the reduction in computational complexity. The multiplication of the matrix is a space of $N_{s}\;X\;N_{t}^{2}$, and the complexity of the simple deep learning procedure is $O\left(L^{2}N_{s}N_{t}^{2}\right)$ and for our proposed DDN method is $O\left(LN_{s}N_{t}^{2}\right)$. This order is very less than the conventional methods of HBF [19,27,28]. To substantiate the low computationalcomplexity of the DDN scheme by instinct, we defineKas the number of paths as well as the computational complexity of the method proposed is presented and that of other traditional approaches to precoding, which is shown in TABLE-1.

● Algorithm 2: DDN based Hybrid Precoding with Millimeter Wave Massive-MIMO.

**Table 1:** Complexity Order of Proposed and Conventional methods.

Methods	Computational Complexity
Proposed DDN	$O\left(LN_{s}N_{t}^{2}\right)$
Deep Learning base HBF [19]	$O\left(L^{2}N_{s}N_{t}^{2}\right)$
SIC-based precoding [27]	$O\left(2N_{r}N_{s}\left(N_{t}^{2}\left(K+N_{s}N_{r}\right)\right)\right)$
Spatially sparse precoding [28]	$O\left(2N_{r}^{3}\left(N_{r}^{4}N_{t}+N_{r}^{2}L^{2}+N_{r}^{2}N_{t}^{2}L\right)\right)$

Inputs: AOA $\theta_{p}^{r}$ and AOD $\theta_{p}^{t}$ (Physical).

Output: Optimized hybrid precoderG_1_.

1. Initialization: Iteration cost is initialized as j = 0 and the weight is w = 0. Initialize the error threshold, meanwhile, as ñ = 10-7. In addition, we set G RF = 0 and G BB = 0.τ = 10-7. Furthermore, we set G_RF = 0 and G_BB = 0.
2. Create a sequence for training and also randomly generated series are $\theta_{p}^{r}$ and $\theta_{p}^{t}$.
3. Build a new approach called DDN.
4. Simulate a channel in wireless manner with synthetic deformation/noise by processing the simulator.
5. while (error ≥ τ).
6. Obtain $G_{RF}$ using Algorithm 1 and train the Deep Net-A.
7. Obtain $G_{BB}$ using GSP and train the Deep Net-D.
8. For obtained $G_{RF}andG_{BB}$ from dual network, processing the SGD with momentum.
9. Update $G_{RF}andG_{BB}$ according to Eq. (26).
10 Obtain the bias between G_1_ and $G_{RF}G_{BB}$, in which it is acquired from the network's output layer w.r.t Eq. (27).
11. end while.

## 3 Results and discussion

To demonstrate the effectiveness of the proposed Hybrid Beamforming (HBF) design in the unconstrained case of mmWave massive MIMO system, we evaluate the BER, Spectral Efficiency, Energy Efficiency and Achievable Capacity as data rate for codebook size of 32 and 64 configurations. We analyzed the performance of DDN with the different parameters using MATLAB simulation of version 2018a. The simulation configurations are mentioned in the below Table 2.

**Table 2:** DDN Simulation Parameters configurations.

Parameter	Value
Modulation Type	BPSK
Number of Data symbols	32
Ntx	32
Nrx	16
Number of RF chain	4
Number of Phase shifter Bits	7
Number of paths	3
Transmit Power	15 dBm
Carrier Frequency	28 GHz
Channel Bandwidth	1 GHz
Channel Sampling Rate	100 Msps
Number of Iterations	4500
SNR Range	0–40 dB
Spacing distance	λ/2

**Fig. 4:**
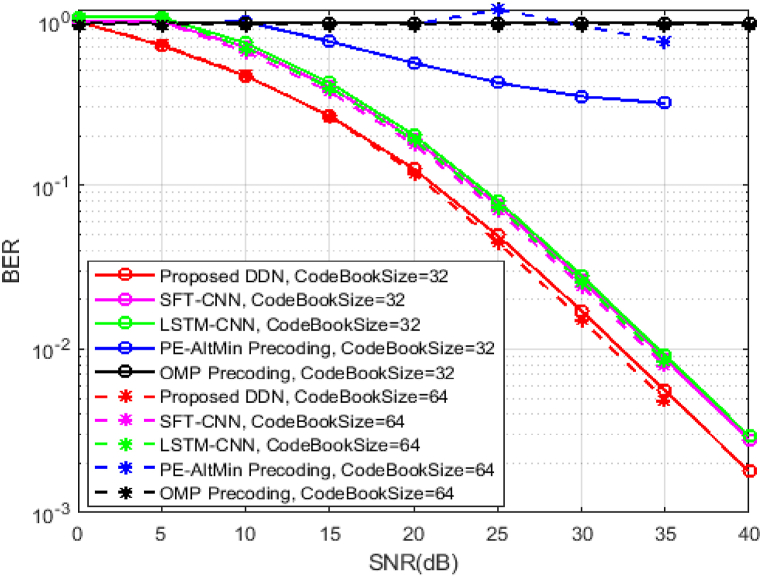
BER curve Vs SNR for different Hybrid Beamforming methods.

The Bit Error Ratio concert of the DDN methodology contrasted to SFT-CNN assisted hybrid precoding scheme [29], the LSTM–CNN–based precoding method [30], the OMP precoding method [5], and the PE-AltMin-based precoding scheme [7] are examined to assess the dominance of the proposed approach. As indicated in Fig. 4, the dual method based on deep learning outperforms conventional schemes. In addition, the improvement in performance between the proposed DDN and the traditional procedures are more apparent, which is accredited to deep learning's excellent representational ability. In addition, since the DDN takes advantage of the structural information and can advance every epoch of the proposed hybrid precoding algorithm and verified the Millimeter-Wave massive MIMO approach is superior to the precoding mean based on deep learning, implying that with the help of deep learning, the current non-convex optimization in hybrid precoding can be solved.

As of now, there is no analytical upper bound on how spectrally efficient of mmWave Massive MIMO system will change according to more number of antennas. So, first of all we need to set some reasonable limit. A basic formula for the spectral efficiency calculation is:

(29) $$S_{e}=U.\left(1-\frac{U}{\tau_{c}}\right).\left(1+\frac{Q_{CSI}.Tx.\frac{\rho }{U}}{\rho +1}\right))$$

Where Tx is the transmission antennas number U is the space multiplexed users number, Q_CSI_ is the quality of the channel assessment and τ_c_ is the channel usage number per channel consistent block. The variable ρ is the user's nominal signal-to-noise (SNR) ratio, obtained when M = K = 1. Eq. Eq. (18) is a thorough lower limit of sum capacity achieved by maximum precoding ratio, i.e. fading channels of Rayleight and equal allocation of power.

**Fig. 5:**
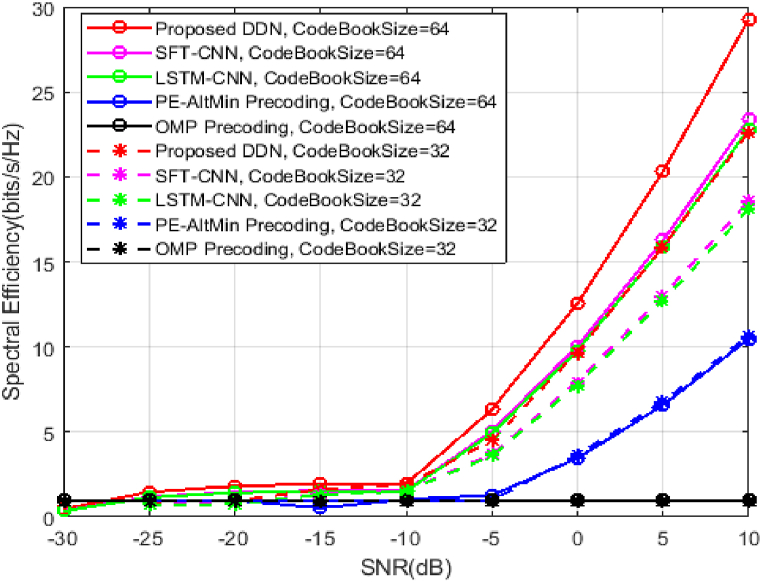
Spectral Efficiency curve Vs SNR for different Hybrid Beamforming methods.

Fig. 5, illustrates the performance of the SE against the DDN-based hybrid precoding scheme of the SNR [29], LSTM–CNN–based precoding scheme [30]. As described in Fig. 5 as the SNR increases in all systems, the proposed approach examines the civilization of spectrum efficiency.

Results are compared the performance of SE for two different codebook size values of 32 and 64. As codebook size increases the SE of the system getting increased. It can be seen, too, from Fig. 5 that other approaches are outperformed by the proposed hybrid precoding system, which delivers better hybrid precoding performance for excellent mapping and deep learning capabilities. Moreover, as the SNR increases, the deep learning system's performance gap and that of other approaches increases. The efficacy of the suggested hybrid precoding scheme is further demonstrated by this superior performance.

**Fig. 6:**
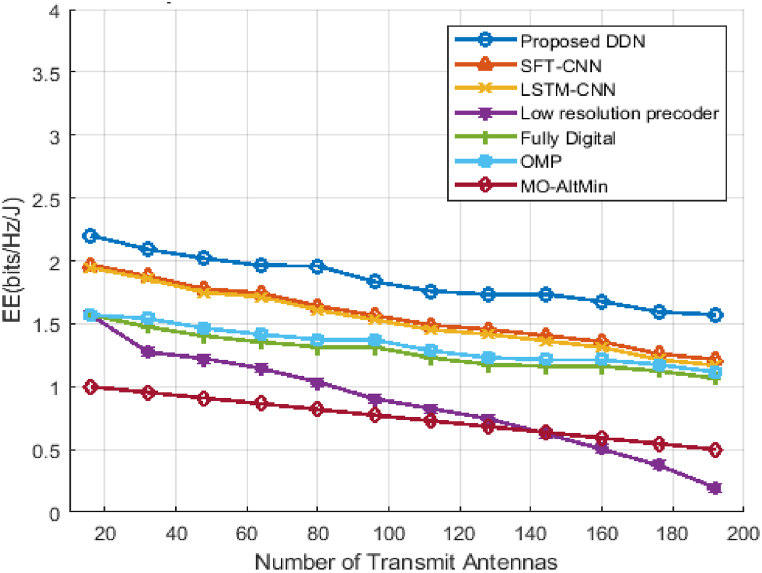
Energy Efficiency curve Vs different number of antennas Hybrid Beamforming methods.

The above Fig. 6 shows the EE effect for the number of transmitting antennas when the SNR is set to 15 dB. The current OMP and MO-Alt Min beamforming algorithms use six RF chains in the simulation. The figure shows the proposed DDN hybrid precoding is higher than the known literary solutions [5,7,[27], [28], [29], [30]]. The figure shows that the EE is reduced linearly with an increasing number of antennas. The DDN proposed improves EE with optimal D-A precoders in the system. The number of transmission antennas is the same with a fully digital system as the number of RF chains, which reduces EE performance, as the RF chain uses more power when the number of antennas increases. In both hybrid precoding, OMP and MO-Alt Min lose EE linearly because the number of RF chains has not been optimized. The performance of SFT-CNN and LSTM-CNN is more or less equal to that of DDN.

**Fig. 7:**
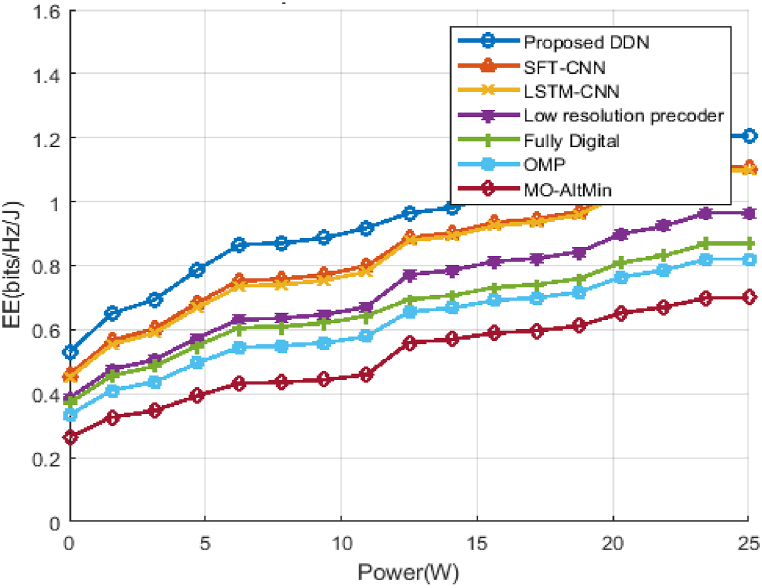
Energy Efficiency curve Vs Transmit Power.

In Fig. 7, we have shown EE performance for various transmitting power levels. As the power increase the EE of the system is generally reduced because of the number of RF chains we used. Although with our proposed DDN method we have achieved the highest EE in RF chain use optimization as well as a reduction in precoding complexity than all existing methods. The achievable capacity is close to the completely digital system and the total power is optimized, which equates the EE performance with the energy consumption. In comparison to the fully digital beamformer, the EE receives 0.4Kbit/Hz/J.

**Fig. 8:**
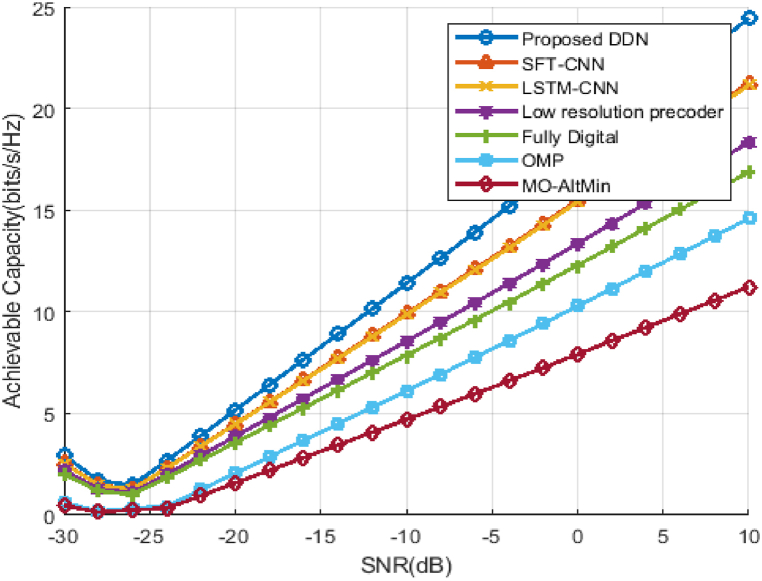
Achievable capacity of hybrid D-A mm-Wave system.

The above figure, Fig. 8 shows the capabilities for the hybrid D-A mm-Wave system can be achieved with 8 plots, if N RF^t^ = N RF^r^=N s, as SNR function. We've taken SNR range here from −30dB to 10 dB. To show the efficiency of the data rate, we consider the lowest SNR values and from Fig. 8 it is observed that the system capacity increases as the SNR improves. The capacity of our proposed DDN precoder is more than the methods SFT-CNN and LSTM-CNN and almost 10 Kbits/s/Hz than the performance of the Fully Digital Method. This is because DDN can precisely design the optimal precoders that capitalize on the system's achievable capacity and, therefore, exceed existing algorithms.

**Fig. 9:**
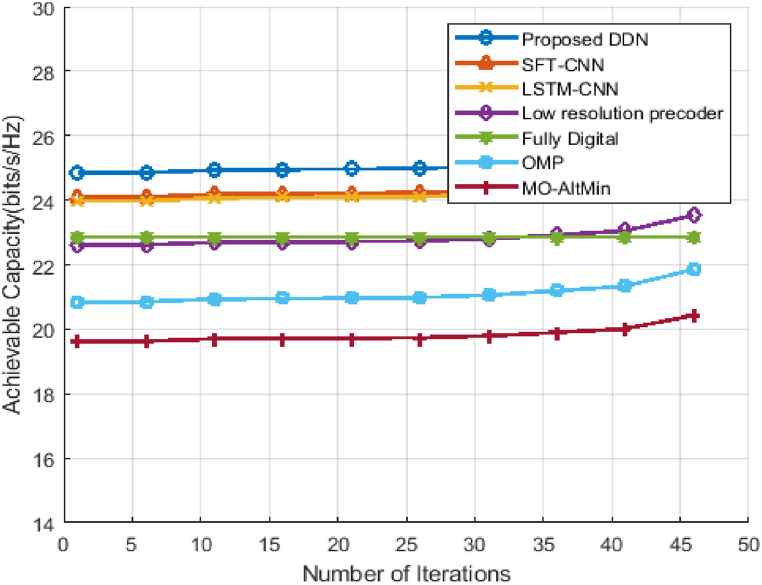
Convergence of Achievable capacity for hybrid D-A mm-Wave system.

Fig. 9 shows the convergence behavior of all the algorithms which we considered for the analysis of the proposed DDN hybrid D-A system performance with SNR = 0 dB. The achievable capacity achieved is averaged over 4500 independent channel generations. As depicted from Fig. 9, the proposed algorithm converges within 5 iterations itself which proves that the DDN provides the high data rate too fast than existing methods. Utilizing the pre-trained network for optimization of hybrid beamforming, it improves the convergence speed to a steady-state value. At the same time, remaining existing methods converged after the 20 iterations only. SFT-CNN, LSTM-CNN, OMP methods converged at 25, 27 and 43 iterations respectively.

**Fig. 10:**
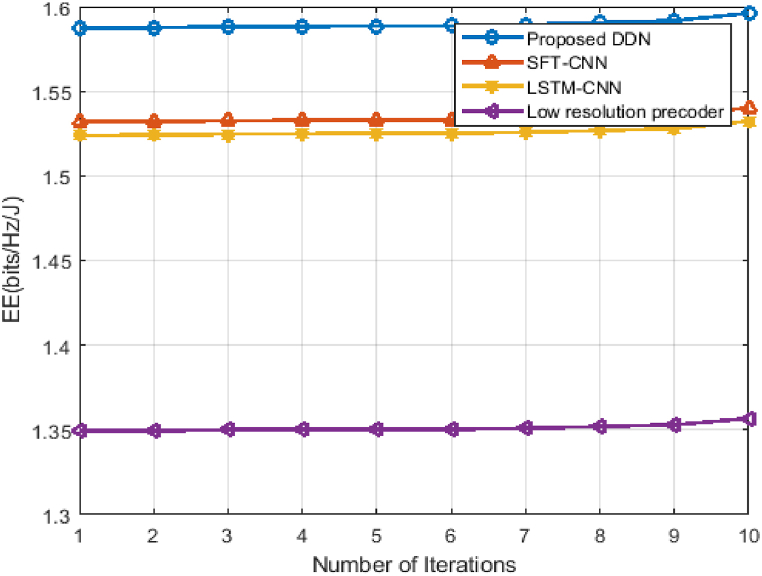
Convergence of EE for hybrid D-A mm-Wave system.

Fig. 10 illustrates the performance of EE convergence for proposed DDN and comparing it with existing nearly close algorithms of SFT-CNN, LSTM-CNN and low resolution precoder. For an efficient system, we have to search for optimal precoding and combining matrix in the process of hybrid beamforming. Using DDN we can be able to achieve the high EE values but also, we have constrained in terms of fastest prediction. The processing delay has to be achieved as low as possible, we reach the optimal system. By the way line of this methodology, we are shown that DDN converged at 3 iterations with EE of 1.59Kbits/Hz/J.

SFT-CNN and LSTM-CNN methods converged at 5th iterations also, but it cannot be able to reach the value of DDN. LR precoder convergence is much slower than these three methods. So, it shows that the fastest optimization is achieved in our DDN.

The spectral efficiency will rise logarithmically if N_t_ is raised while U is fixed. Because this is what analog beamforming can accomplish for U = 1, we are concerned that the industry may be misled by the benefits that 5G beamforming would provide. The spectral efficiency will rise linearly with the number of users if N_t_ and U are increased simultaneously to maintain a fixed N_t_/U ratio. The same power transmission is divided among U users; however, the power reduction per user is compensated for by raising the array gain N_t_. As a result, the performance is the same for each user.

**Fig. 11:**
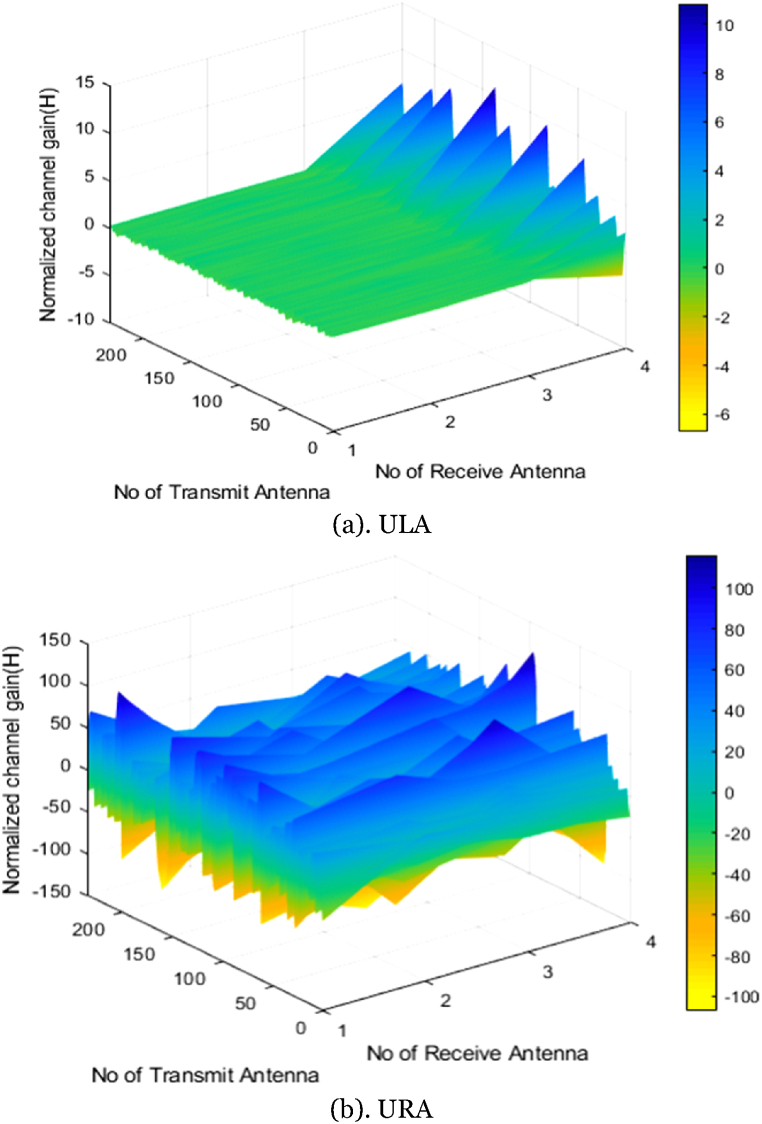
Normalized channel gain of ULA and URA

We will get a significant gain from spatial multiplexing if we enlarge the user base. The highest advantages undoubtedly result from spatial multiplexing, and increasing the number of antennas is a must to enable such multiplexing. Fig. 11(a and b) depicts the array gain of ULA and URA correspondingly, which provides the noticeable difference of highest gain in URA than ULA.

## 4 Conclusions and scope

In this paper, we proposed an efficient algorithm for boosting the hybrid precoding performance, including reduces the processing difficulties and influencing the relational information of mmWave massive MIMO system. We derived the algorithm of dual deep-learning-network based multi resolution code book for hybrid beam forming method. We evaluated the proposed approach DDN based method effectiveness and enlightening the DDN can ease the hybrid-precoding logic because of its vast detection and planning capacities. The suggested DDN-based MR code book hybrid beam forming system provides support for high angular resolution, in contrast to existing techniques. We provide Deep learning architecture, which gets the precoded result from analog and digital precoders and performs the beam training method, in order to address the issue of the suggested DDN based hybrid beam forming system's use of a multi resolution code book. In comparison to traditional methods, the DDN structure of the multi-resolution code book is altered by the use of reference RF beam former. We can see from the performance study that the new method, DDN, produced more useful findings than the previous ones. With 7Kbits/s/Hz and 1e-1, respectively, the key metrics of spectral efficiency and BER are enhanced. We have also demonstrated how the performance of the suggested scheme gradually improves as the number of training epochs rises, until settling at a saturated value. Also, at the receiver reconstruction section, we are achieving the high beam forming gain. Future work can be expanded by concentrating on beam alignment problem optimization, which will further boost system data rate for a 6G network.

## Data availability statement

Data sharing is not applicable to this article as no new data were created or analyzed in this study.

## CRediT authorship contribution statement

**Ramesh Sundar:** Writing – review & editing, Writing – original draft, Supervision, Investigation. **Mohammad Amir:** Validation, Software, Methodology, Investigation. **Ranjith Subramanian:** Software, Resources, Formal analysis. **D. Prabakar:** Software, Methodology, Investigation. **Jayant Giri:** Visualization, Validation, Resources, Investigation. **G. Balachandran:** Software, Resources, Investigation. **Furkan Ahmad:** Supervision, Project administration, Investigation, Funding acquisition.

## Declaration of competing interest

The authors declare that they have no known competing financial interests or personal relationships that could have appeared to influence the work reported in this paper.
